# Supplementary material for: Complex‐centric proteome profiling by SEC‐SWATH‐MS
Source: Mol Syst Biol. 2019 Jan 14;15(1):e8438. doi: 10.15252/msb.20188438 (PMC6346213; doi:10.15252/msb.20188438)
Supplement: Supplementary file 8 — Dataset EV7 [file MSB-15-e8438-s008.zip › feature_plots_string/P25788.pdf]

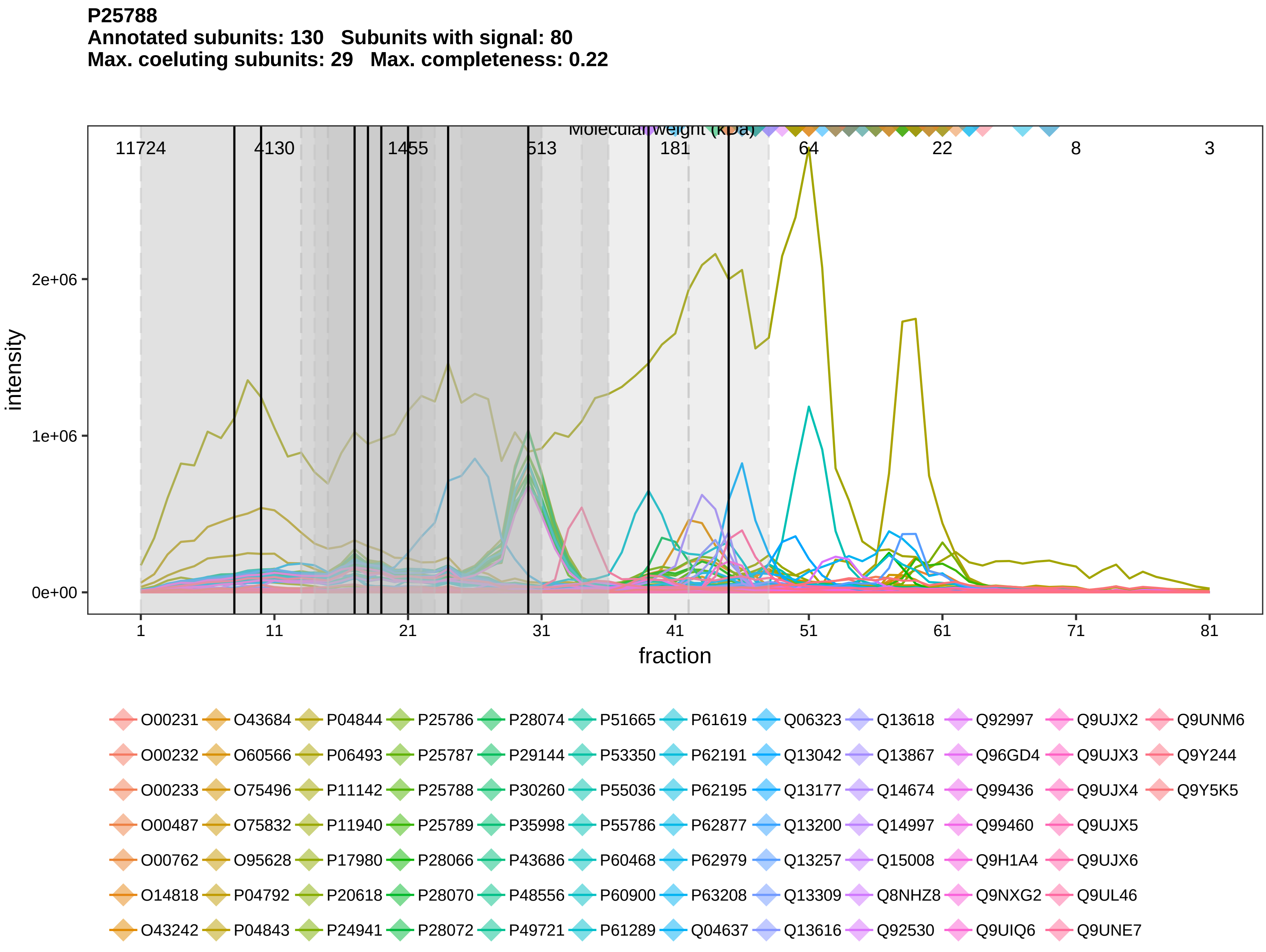
P25788
Annotated subunits: 130 Subunits with signal: 80
Max. coeluting subunits: 29 Max. completeness: 0.22
Molecular weight (kDa)
11724
4130
1455
513
181
64
22
8
3
intensity
2e+06
1e+06
0e+00
fraction
1
11
21
31
41
51
61
71
81
O00231
O43684
P04844
P25786
P28074
P51665
P61619
Q06323
Q13618
Q92997
Q9UJX2
Q9UNM6
O00232
O60566
P06493
P25787
P29144
P53350
P62191
Q13042
Q13867
Q96GD4
Q9UJX3
Q9Y244
O00233
O75496
P11142
P25788
P30260
P55036
P62195
Q13177
Q14674
Q99436
Q9UJX4
Q9Y5K5
O00487
O75832
P11940
P25789
P35998
P55786
P62877
Q13200
Q14997
Q99460
Q9UJX5
O00762
O95628
P17980
P28066
P43686
P60468
P62979
Q13257
Q15008
Q9H1A4
Q9UJX6
O14818
P04792
P20618
P28070
P48556
P60900
P63208
Q13309
Q8NHZ8
Q9NXG2
Q9UL46
O43242
P04843
P24941
P28072
P49721
P61289
Q04637
Q13616
Q92530
Q9UIQ6
Q9UNE7
